# Patterns of First Recurrence and Oncological Outcomes in Locally Advanced Cervical Cancer Patients: Does Surgical Staging Play a Role?

**DOI:** 10.3390/cancers16071423

**Published:** 2024-04-06

**Authors:** Vicente Bebia, Berta Díaz-Feijoo, Álvaro Tejerizo, Aureli Torne, Virginia Benito, Alicia Hernández, Mikel Gorostidi, Santiago Domingo, Melissa Bradbury, Rocío Luna-Guibourg, Antonio Gil-Moreno

**Affiliations:** 1 Gynecologic Oncology Division, Vall d’Hebron Barcelona Hospital Campus, Universitat Autònoma de Barcelona, 08035 Barcelona, Spainmelissa.bradbury@vallhebron.cat (M.B.); antonioimma@yahoo.es (A.G.-M.); 2 Institute Clinic of Gynecology, Obstetrics and Neonatology, Hospital Clinic de Barcelona, Institut d’Investigacions Biomèdiques August Pi i Sunyer (IDIBAPS), Universitat de Barcelona, 08035 Barcelona, Spain; 3 Department of Obstetrics and Gynecology, Hospital Universitario 12 de Octubre, 28041 Madrid, Spain; 4 Department of Gynecologic Oncology, Complejo Hospitalario Universitario Insular-Materno Infantil, 35016 Las Palmas de Gran Canaria, Spain; 5 Department of Gynecology, Hospital Universitario La Paz, 28046 Madrid, Spain; 6 Department of Gynecology and Obstetrics, Hospital Universitario Donostia, 20014 San Sebastián, Spain; mgorostidi@sego.es; 7 Department of Gynecology Oncology, Hospital Universitari i Politècnic La Fe, 46026 Valencia, Spain; 8 Department of Obstetrics and Gynecology, Hospital de la Santa Creu I Sant Pau, Universitat Autònoma de Barcelona, 08041 Barcelona, Spain; ropiluna@gmail.com; 9 CIBERONC Centro de Investigación Biomédica en Red Cáncer, 08193 Madrid, Spain

**Keywords:** cervical cancer, aortic lymphadenectomy, surgical staging

## Abstract

**Simple Summary:**

Whenever cancer of the uterine cervix is diagnosed in a locally advanced stage, it is important to know whether it affects the lymph nodes above the pelvis or not. There are two ways of ruling it out: by a surgery, called paraaortic lymphadenectomy, or by imaging tests. With this study, we wanted to see whether paraaortic lymphadenectomy affected the natural evolution of the tumor by looking at the differences in the recurrence rate between both groups. We used a statistical technique that makes both groups comparable. We observed that patients who underwent a paraaortic surgery suffered more recurrences (both at the lymph nodes and at distance) and survived less than those treated only with the information from the imaging tests.

**Abstract:**

Background: We aimed to determine whether surgical aortic staging by minimally invasive paraaortic lymphadenectomy (PALND) affects the pattern of first recurrence and survival in treated locally advanced cervical cancer (LACC) patients when compared to patients staged by imaging (noPALND). Methods: This study was a multicenter observational retrospective cohort study of patients with LACC treated at tertiary care hospitals throughout Spain. The inclusion criteria were histological diagnosis of squamous carcinoma, adenosquamous carcinoma, and/or adenocarcinoma; FIGO stages IB2, IIA2-IVA (FIGO 2009); and planned treatment with primary chemoradiotherapy between 2000 and 2016. Propensity score matching (PSM) was performed before the analysis. Results: After PSM and sample replacement, 1092 patients were included for analysis (noPALND n = 546, PALND n = 546). Twenty-one percent of patients recurred during follow-up, with the PALND group having almost double the recurrences of the noPALND group (noPALND: 15.0%, PALND: 28.0%, *p* < 0.001). Nodal (regional) recurrences were more frequently observed in PALND patients (noPALND:2.4%, PALND: 11.2%, *p* < 0.001). Among those who recurred regionally, 57.1% recurred at the pelvic nodes, 37.1% recurred at the aortic nodes, and 5.7% recurred simultaneously at both the pelvic and aortic nodes. Patients who underwent a staging PALND were more frequently diagnosed with a distant recurrence (noPALND: 7.0%, PALND: 15.6%, *p* < 0.001). PALND patients presented poorer overall, cancer-specific, and disease-free survival when compared to patients in the noPALND group. Conclusion: After treatment, surgically staged patients with LACC recurred more frequently and showed worse survival rates.

## 1. Introduction

In countries in the in the European Union, more than 28,000 women were estimated to be newly diagnosed with cervical cancer during 2022 [1]. Population-based vaccination and screening [2] programs have been proven to reduce cervical cancer incidence. Consequently, cervical cancer continues to be diagnosed predominantly in low- and middle-income countries [3]. More than half of new cervical cancer diagnoses will be locally advanced stage tumors (IB3, IIA2-IVA FIGO 2018 stages) [4].

Concurrent pelvic radiotherapy and cisplatin-based chemotherapy plus brachytherapy remains the standard treatment for this subset of patients. The extension of the radiotherapy field to include the aortic region is widely accepted for cases with aortic involvement, diagnosed by either imaging or surgery (i.e., extraperitoneal aortic lymphadenectomy up to the renal vein) [5,6].

Although aortic surgical staging does not seem to have prognostic implications on locally advanced cervical cancer (LACC) [7], the biological effects of this intervention on cervical cancer evolution remains unknown. Even though several studies on aortic staging, including one prospective trial, have reported recurrence data, the results encountered are highly heterogeneous. Moreover, gynecologic oncologists around the world are highly concerned about the possible effects that tumor manipulation and spillage may imply in cervical cancer patients. With this goal, we aimed to determine whether surgical staging by minimally invasive paraaortic lymphadenectomy (PALND) affects the pattern of first recurrence in treated LACC patients and modifies the oncological outcomes of this disease.

## 2. Material and Methods

This was a multicenter observational retrospective cohort study of patients with LACC carried out at the Departments of Gynecology of 11 tertiary care hospitals throughout Spain. Specifics on patient inclusion criteria are detailed elsewhere [8]. For this analysis, we included patients with (a) a histological diagnosis of squamous carcinoma, adenosquamous carcinoma, and/or adenocarcinoma (excluding undifferentiated or unknown histology subtypes); (b) patients with FIGO stages IB2, IIA2-IVA, according to FIGO 2009 system [9]; and (c) patients who had received planned treatment with primary chemoradiotherapy (pelvic irradiation field) between 2000 and 2016. The exclusion criteria were defined as an Eastern Cooperative Oncology Group (ECOG) performance status equal or greater than 2, age older than 80 years, or incomplete follow-up.

Two groups were considered for the analysis: patients who received a staging PALND to tailor radiotherapy fields prior to definitive treatment (PALND group) and patients with aortic status determined by imaging techniques exclusively (noPALND group).

The study was approved by the Clinical Research Ethics Committee of Hospital Universitari Vall d’Hebron (study protocol 159/2015) as the reference center and by the Institutional Review Boards of the participating hospitals.

In accordance with the journal’s guidelines, we will provide our data for the purposes of additional data analysis or for the reproducibility of this study in other centers if such data are requested.

### 2.1. Aortic Lymph Node Assessment and Primary Treatment

Aortic node involvement was determined at diagnosis either by surgical aortic staging or clinical staging. When surgically staged, patients underwent a paraaortic lymphadenectomy (PALND) using a minimally invasive extraperitoneal approach. Depending on local protocols, pelvic lymph node debulking of enlarged pelvic nodes (≥20 mm on preoperative imaging tests) was considered. Minimally invasive extraperitoneal PALND for LACC aortic staging has been described extensively by our group before [10].

For clinical staging, aortic status was determined by either positron emission tomography–computed tomography (PET/CT) and/or magnetic resonance imaging (MRI). Aortic lymph nodes were considered as involved when the short axis was greater than 10 mm in MRI or when radiotracer uptake was moderate or high in PET/CT.

Standard treatment consisted of external-beam radiation therapy (EBRT), with a 45 Gy total dose delivered in 25 fractions over 5 weeks. Concurrent weekly cisplatin (40 mg/m^2^) was administered. Either pulse-dose-, high-dose-, or low-dose-rate brachytherapy was administered in more than 90 percent of patients, with a 30–35 Gy dose to point A.

Due to the recruitment period of the study (2000–2016), the FIGO 2009 staging classification was considered rather than the currently used FIGO 2018. Nevertheless, nodal status was reported separately.

### 2.2. Recurrence Diagnosis

After primary treatment, clinical evaluation of tumor response was performed both at 3 and 6 months. Recurrence of the tumor was defined as any finding suspicious for malignancy, as assessed by a multidisciplinary tumor board and/or confirmed by pathology if amenable for biopsy. Due to the long period encompassed by this study and its retrospective nature, several imaging methods (computed tomography [CT], MRI, and PET/CT) were employed.

For this study, only first recurrences were considered. Local relapse was defined as any recurrence in the cervix or body of the uterus or surrounding tissues. Regional or nodal relapse was considered as a recurrence in the pelvic or aortic nodal area. Distant metastasis was defined as any recurrence in any site other than local or regional recurrence. If a patient relapsed at different locations simultaneously, every recurrence was considered independently.

When analyzing regional relapses distribution, three different patterns were considered depending on the location of the recurrence. Therefore, regional relapses were grouped as exclusively pelvic, exclusively aortic, and simultaneously aortic and pelvic.

Disease-free survival (DFS) was defined as time from end of treatment to diagnosis of recurrence. Overall survival (OS) was defined as time from the end of treatment to date of death or last follow-up. Cancer-specific survival was defined as the time from the end of treatment to the date of death from the disease, its treatment, or last follow-up.

### 2.3. Statistical Analysis

Continuous variables are expressed as mean and standard deviation (SD) or as median and interquartile range (IQR) depending on the distribution of the variable and were compared using Student’s *t* test or the Mann–Whitney U test, as appropriate. Categorical variables are expressed as frequencies and percentages and were compared using the χ^2^ test or the Fisher exact test. All tests were 2-tailed. The imputation of missing values was not performed.

Due to the non-randomized nature of our study, propensity score matching was performed before the analysis following the nearest-neighbor method. Variables with less than 5% of missing values were considered. The following variables were selected for matching: age at diagnosis, tumor maximum diameter, grouped FIGO stage, aortic radiotherapy planning, total radiotherapy dose, and treatment days. NoPALND patients were matched to those who received staging PALND on a 1:1 basis with sample replacement, and a caliper width 0.05 standard deviations was selected. Sample replacement consists of a statistical method of balancing both control and experimental arms. It is performed by considering any matched patients several times in the smallest group so that both arms are balanced.

Oncologic outcome was analyzed using the Kaplan–Meier method and the log-rank test. The R statistical program (R Foundation for Statistical Computing, Vienna, Austria) was used for data analysis.

## 3. Results

A total of 922 patients were included for primary analysis (noPALND n = 288, PALND n = 634). Demographic and tumoral variables were compared between both groups. Patients in the PALND group were significantly younger, had earlier stage tumors, and were less likely to be treated with extended-field radiotherapy (Table 1). Ninety-seven percent of patients in the surgically staged group underwent total PALND (up to the left renal vein). As previously reported by our group, a median of 13 nodes (IQR: 9-17) were excised in these patients.

After PSM, a total of 1092 patients were included for analysis (noPALND n = 546, PALND n = 546). Both groups were then comparable regarding all basal (demographic and tumoral) and treatment variables considered (Table 1).

The median age was 48 years. Sixty-eight percent of patients were diagnosed with FIGO 2009 stages IIB or higher. Most patients had squamous carcinomas, while 14.9% corresponded to adenocarcinomas, 1.5% to adenosquamous tumors, and the remaining 2.1% to other histology variants. Less than one third of the patients had nodal involvement at diagnosis.

Although significantly different among groups after PSM, the median radiotherapy dose was 45 Gy for both groups. Ninety-one percent of patients received brachytherapy as part of the treatment. Seventeen percent of all patients were planned to receive extended-field radiotherapy. (Table 1).

Twenty-one percent of patients recurred during follow-up, with the PALND group accounting for 28% and the noPALND group for 15% (*p* < 0.001). Local recurrence rates were similar among groups, corresponding to 8.7% of the patients (noPALND: 8.6%, PALND: 8.8%, *p* = 1).

**Figure 1:**
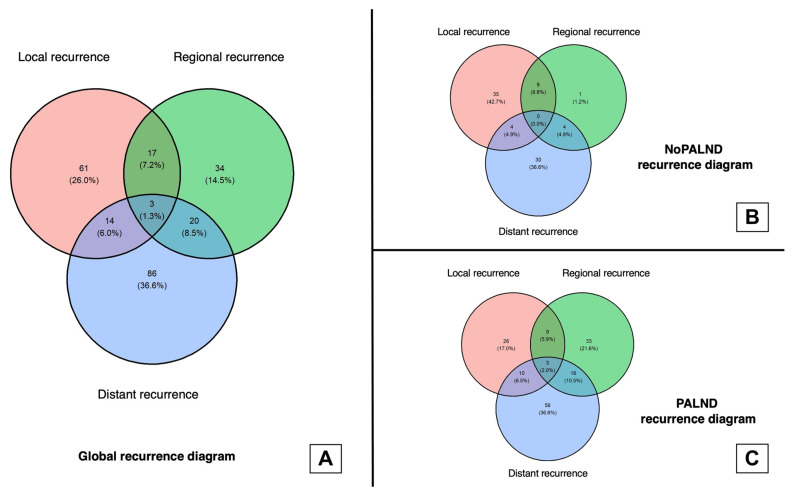
Venn diagrams on distribution of first recurrences. (**A**): Venn diagram on distribution of first recurrences in the whole cohort. (**B**): Venn diagram on distribution of first recurrences in the noPALND group. (**C**): Venn diagram on distribution of first recurrences in the PALND group.

More regional (nodal) recurrences occurred in the PALND group (noPALND: 2.4%, PALND: 11.2%, *p* < 0.001). Among those who recurred regionally, 57.1% recurred only at the pelvis, 37.1% recurred only at the aortic nodes, and 5.7% recurred simultaneously at both the pelvic and aortic nodes. Differences in the distribution of nodal recurrences were observed between the PALND and no PALND patients as, when analyzed separately, both pelvic (noPALND: 1.3%, PALND: 6.8%, *p* < 0.001) and aortic (noPALND: 0.9%, PALND: 4.6%, *p* < 0.001) nodal recurrences were more frequently observed in the PALND group (Table 2, Figure 1).

Patients who underwent a staging PALND were more frequently diagnosed with a distant recurrence (noPALND: 7.0%, PALND: 15.6%, *p* < 0.001). The most frequent site for distant recurrence in both groups was the lung (NoPALND: 14/38; PALND: 33/85). The second most frequent site for distant metastasis in noPALND patients were the supradiaphragmatic nodes (11/38), and in the PALND group it was the peritoneum (23/83). No patients recurred peritoneally in the noPALND group (Table 2).

**Figure 2:**
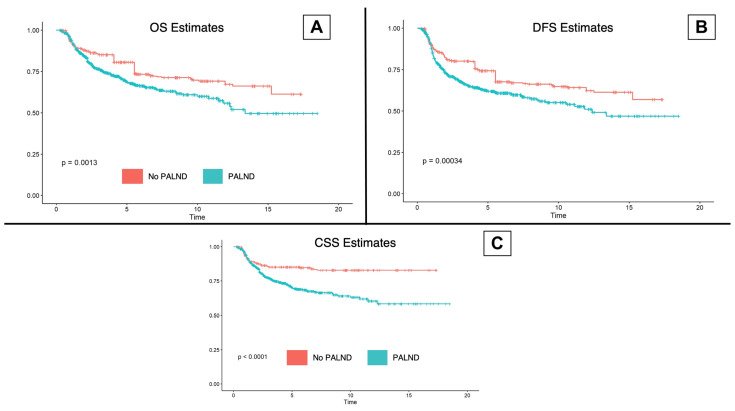
Kaplan–Meier estimates for oncological outcomes. (**A**): Kaplan–Meier estimates for overall survival (OS). (**B**): Kaplan–Meier estimates for disease-free survival (DFS). (**C**): Kaplan–Meier estimates for cancer-specific survival (DFS).

For the whole matched sample, at 5 years, DFS was estimated to be 67.9% (CI 95%: 65.0–71.0), while 74.6% (CI 95%: 71.7–77.5) were estimated to be alive. Cancer-specific survival at 5 years of follow-up was estimated to be 77.6% (CI 95%: 74.9–80.4). The PALND patients presented poorer overall (noPALND: 80.7%, PALND: 68.9%, *p* < 0.001), cancer-specific (noPALND: 85.1%, PALND: 70.3%, *p* < 0.001), and disease-free survival (noPALND: 74.3%, PALND: 61.9%, *p* < 0.001) when compared to the patients in the noPALND group (Figure 2). Median follow-up was 12 years. Oncological outcomes by FIGO stage are available as Supplementary Data S1.

## 4. Discussion

### 4.1. Summary of Main Results

In this study, after controlling for confounding variables by PSM, LACC patients who underwent PALND for aortic staging recurred more frequently regionally and at distant localizations than those clinically staged. No difference in regional recurrence distribution was observed between PALND and noPALND patients. The PALND patients recurred more frequently and presented poorer overall, cancer-specific, and disease-free survival than the noPALND patients.

### 4.2. Results in the Context of the Published Literature

Patterns of recurrence have been described thoroughly in the literature for local, regional, and distant localizations [7,11,12,13]. However, to our knowledge, a well-designed study specifically addressing the effect of surgical staging on pattern of recurrence has not been previously performed.

Approximately one third of the patients treated for cervical cancer will recur during follow-up [11]. We observed a similar rate of recurrence in our patients. However, there is contradictory data on patterns of recurrence throughout the literature. In our study, both local and distant recurrences were recorded for two thirds of the patients, while approximately only one third of them recurred at the pelvic or aortic nodes. Most studies describe recurrences at distant sites [7,12,13]. In the UTERUS-11 trial, Marnitz et al. observed that nearly 90% of both the surgically and clinically staged groups recurred distantly [7]. Gennigens et al. found that, in their treated LACC patients, the sites of disease at the time of first relapse were most frequently distant in two thirds of their patients, while the sites were local in only one third of patients [13]. Heterogeneity on reporting data, as well as the large period encompassed by most studies, may explain the differences across the literature.

Regarding distant recurrence patterns, in our study, peritoneal carcinomatosis was not found in the noPALND group, while it was the second most common site for distant recurrence in the PALND group. One possible explanation for this could arise from performing a peritoneal window in extraperitoneal PALND to diminish lymphocyst occurrence. This could have put malignant cells harbored in the aortic or pelvic nodes in contact with the peritoneal cavity, then resulting in peritoneal implants. Although we are in no place to establish a causal association between PALND and peritoneal carcinomatosis, this association should be further investigated.

As previously reported by our group [8] and others [7], up to one third of LACC patients may change their initial clinically staged treatment plan regarding aortic irradiation after staging PALND. Despite the clear implications that surgical staging may have in radiotherapy treatment planning, staging PALND has shown uneven prognostic results. In our analysis, we found that, after PSM, patients who underwent PALND presented poorer survival rates when compared to those clinically staged. In the first randomized clinical trial to evaluate PALND, Lai et al. [14] reported worse overall survival in patients undergoing staging PALND. As this publication was received with much criticism by the gynecologic oncology community (given the premature cessation of the trial, the unreported rates of extended field radiotherapy, the unpowered sample, and the poorer prognostic factors in the surgically staged arm), other authors, including our group, retrospectively reviewed their data during the following 20 years [8,15,16,17], with no clear conclusions on whether performing a staging PALND had a survival benefit or not.

In their recently published randomized controlled trial, UTERUS-11, Marnitz et al. [7] were not able to show a significant prognostic benefit of staging PALND when compared to clinically (MRI or CT) aortic staged patients. Nevertheless, it is stated that surgical aortic staging may have some prognostic influence, as cancer-specific survival (post hoc analysis) seemed to benefit staging PALND, while patients with parametrial involvement (FIGO IIB) seemed to benefit from surgical staging in terms of DFS (HR 0.51, 95% CI 0.30 to 0.86). As shown in the Supplementary Data S1, in our study, we found no significant differences between both groups in FIGO IIB patients. Nevertheless, our study was neither designed nor powered to evaluate this outcome.

### 4.3. Strengths and Weaknesses

The retrospective nature of this study may have limited our ability to detect differences in patterns of recurrence. Additionally, despite making our results more robust, PSM relies on variables with less than 5% of missing values. Some variables (such as time from diagnosis to starting radiotherapy) were not considered due to their high missing values rate, and this may have led to selection bias. Due to the fact that the patients included were treated in different centers across a long period of time, we found heterogeneity in the imaging techniques used for clinical staging, which could have affected how these patients were planned for aortic radiotherapy treatment.

This is the first study to address whether surgical staging in LACC patients affects patterns of recurrence and observe an association between surgical staging and total, regional, and distant recurrence. Future knowledge on patterns of recurrence may improve our ability to predict, detect, and treat LACC patients who will experience a relapse during follow-up.

### 4.4. Implications for Practice and Future Research

As seen in our study, more distant and regional recurrences were observed in patients undergoing surgery for aortic staging. While isolated regional recurrences were rarely seen in noPALND patients, more than one third of patients who recurred regionally in the PALND group did not have any evidence of concurrent relapses. In our opinion, one possible explanation for this observation could be that positive nodes may be manipulated or even torn during staging surgery. Because this study encompasses a high number of years (2001–2016), it is possible that surgical advances in the PALND technique (mostly described during the last 20 years [18]) and an awareness of surgical safety measures in minimally invasive oncological surgeries were not present throughout all of the study period.

With that in mind, the distribution of distant metastasis showed differences between surgical or clinically staged patients. While peritoneal carcinomatosis was not observed as a recurrence site in the noPALND group, it was the second most frequent site at which distant recurrence in the PALND group was observed. Peritoneal spillage has been addressed by many authors during the last five years as a possible cause for postoperative carcinomatosis after cervical cancer surgery [19,20]. Again, manipulation and tearing of positive nodes may lead to peritoneal spillage in the presence of a peritoneal window, favoring the peritoneal implants. More studies should be performed to properly address this relevant issue.

On the other hand, PET/CT diagnostic accuracy may decrease when pelvic nodal uptake is observed but no aortic involvement is determined. As recently reported by Gouy et al. [21] and Martinez et al. [22], false-negative rates of PET/CT for diagnosing aortic involvement when pelvic nodes seem affected may range between 17 and 27%. Therefore, surgical staging seems to be justified in this subgroup of patients.

The survival benefit of aortic staging remains unknown. The one and only properly designed randomized controlled trial failed to show a positive difference when comparing surgical to clinical aortic staging in LACC patients [7]. Nevertheless, this study did not use PET/CT as a staging procedure and was not designed to answer whether patients with pelvic positive nodes and negative aortic nodes in PET/CT scans would benefit from this strategy. The upcoming PAROLA trial, an international multicenter trial designed with the aim to evaluate a disease-free survival benefit in the clinical IIIC1 (as per PET/CT) setting may bring some light to this question [23].

## 5. Conclusions

After LACC treatment, in our retrospective study, we found that surgically staged patients with PALND experienced more recurrences when compared to clinically staged patients, specifically in regional and distant localizations. In addition, surgically staged patients showed poorer oncological outcomes.

Future clinical trials may elucidate whether surgical staging plays a role in patients with pelvic-positive, aortic-negative nodes in PET/CT.

## Figures and Tables

**Table 1:** Before and after propensity score matching demographic and oncologic variables’ distributions.

	Before PSM				After PSM			
Variable	No PALND (n = 288)	PALND (n = 634)	Total (n = 922)	*p* Value	No PALND (n = 546)	PALND (n = 546)	Total (n = 1092)	*p* Value
	Median (IQR) or n(%)				Mean (SD), Median (IQR) or n(%)			
Age (years)	54 (45–61)	49 (41–58)	51 (42–60)	**<0.001**	47 (43–61)	49 (41–59)	48 (41–60)	0.493
Size (mm)	50 (44–65)	50 (40–55)	50 (40–60)	**<0.001**	50 (35–60)	50 (40–57)	50 (40–60)	0.754
Histological subtype				0.690				0.094
Squamous	223 (77.4)	514 (81.1)	737 (79.9)		447 (81.9)	442 (81.0)	889 (81.4)	
Adenocarcinoma	48 (16.7)	90 (14.2)	138 (15.0)		86 (15.8)	77 (14.1)	163 (14.9)	
Adenosquamous	8 (2.8)	15 (2.4)	23 (2.5)		3 (0.5)	13 (2.4)	16 (1.5)	
Undifferentiated	7 (2.4)	10 (1.6)	17 (1.8)		7 (1.3)	10 (1.8)	17 (1.6)	
Other	2 (0.7)	5 (0.8)	7 (0.8)		3 (0.5)	4 (0.7)	7 (0.6)	
FIGO 2009 Staging				**<0.001**				0.845
IB2—IIA2	38 (13.2)	187 (29.5)	225 (24.4)		173 (31.7)	169 (31.0)	342 (31.3)	
IIB—IVA	250 (86.8)	447 (70.5)	697 (75.6)		373 (68.3)	377 (69.0)	750 (68.7)	
Nodal status				0.171				0.199
N0	183 (63.8)	422 (67.1)	605 (66.0)		351 (64.3)	378 (69.2)	729 (66.8)	
N1	100 (34.8)	189 (30.0)	289 (31.6)		177 (32.4)	150 (27.5)	327 (29.9)	
Nx	4 (1.4)	18 (2.9)	22 (2.4)		18 (3.3)	18 (3.3)	36 (3.3)	
Radiotherapy initial volume				**<0.001**				0.936
Pelvic	118 (41.0)	489 (77.1)	607 (65.8)		450 (82.4)	452 (82.8)	902 (82.6)	
Pelvic and Aortic	167 (58.0)	107 (16.9)	274 (29.7)		96 (17.6)	94 (17.2)	190 (17.4)	
Unknown	3 (1.0)	38 (6.0)	41 (4.5)		NA	NA	NA	
Total dose (Gy)	45 (45–46)	45 (45–50)	45 (45–50)	0.201	45 (45–46)	45 (45–50)	45 (45–47)	0.007
Length of treatment (days)	37 (35–42)	38 (35–44)	38 (35–43)	0.376	38 (34–43)	38 (35–44)	38 (35–43)	0.154
Brachytherapy				0.196				0.507
Yes	255 (88.5)	580 (91.5)	835 (90.6)		498 (91.2)	505 (92.5)	1003 (91.8)	
No	33 (11.5)	54 (8.5)	87 (9.4)		48 (8.8)	41 (7.5)	89 (8.2)	

Abbreviations: PSM: propensity score matching. PALND: paraaortic lymph node dissection. NA: not applicable. Gy: gray. Bold: statistically significant.

**Table 2:** Distribution of recurrences and oncological outcomes.

	No PALND (n = 546)	PALND (n = 546)	Total (n = 1092)	*p* Value
Any recurrence	82 (15.0)	153 (28.0)	235 (21.5)	**<0.001**
Local recurrence	47 (8.6)	48 (8.8)	95 (8.7)	1
Regional recurrence	13 (2.4)	61 (11.2)	74 (6.8)	**<0.001**
Pelvic recurrence	7 (1.3)	37 (6.8)	44 (4.0)	**<0.001**
Aortic recurrence	5 (0.9)	25 (4.6)	30 (2.7)	**<0.001**
Patterns of regional recurrence				
Exclusively pelvic recurrence	7 (58.3)	33 (56.9)	40 (57.1)	**1**
Exclusively aortic recurrence	5 (41.7)	21 (36.2)	26 (37.1)	
Pelvic and aortic concomitant recurrence	0 (0.0)	4 (6.9)	4 (5.7)	
Distant recurrences	38 (7.0)	85 (15.6)	123 (11.3)	**<0.001**
Lung/Pleura	14	33	47	
Liver	7	20	27	
Supradiaphragmatic lymph nodes	11	14	25	
Peritoneum	0	23	23	
Bone	1	12	13	
Psoas muscle (LND surgical bed)	0	4	4	
Abdominal Wall	0	1	1	
Inguinal lymph nodes	0	2	2	
Adrenal gland	6	1	7	
Brain	0	2	2	
5-year overall survival (%, CI 95%)	80.7 (77.1–84.4)	68.9 (64.6–73.4)	74.6 (71.7–77.5)	**<0.001**
5-year cancer-specific survival (%, CI 95%)	85.1 (81.9–88.4)	70.3 (66.1–74.8)	77.6 (74.9–80.4)	**<0.001**
5-year disease-free survival (%, CI 95%)	74.3 (70.4–78.4)	61.9 (57.6–66.5)	67.9 (65.0–71.0)	**<0.001**

Abbreviations: PALND: paraaortic lymph node dissection. CI: confidence interval. Bold: statistically significant.

## Data Availability

The data of this study are available from the corresponding author upon request.

## Supplementary Materials

The following supporting information can be downloaded at: https://www.mdpi.com/article/10.3390/cancers16071423/s1, Data S1: Oncological outcomes: Overall, cancer specific and disease free survival analysis by FIGO stage (Stage IIB, III).
