# Supplementary material for: Patterns of First Recurrence and Oncological Outcomes in Locally Advanced Cervical Cancer Patients: Does Surgical Staging Play a Role?
Source: Cancers (Basel). 2024 Apr 6;16(7):1423. doi: 10.3390/cancers16071423 (PMC11011205; doi:10.3390/cancers16071423)
Supplement: Supplementary file 1 [file cancers-16-01423-s001.zip › cancers-2891962-supplementary.pdf]

Supplementary material

Oncological outcomes: Overall, cancer specific and disease free survival analysis by FIGO stage (Stage IIB, III).

1. FIGO IIB

1.1. Overall survival

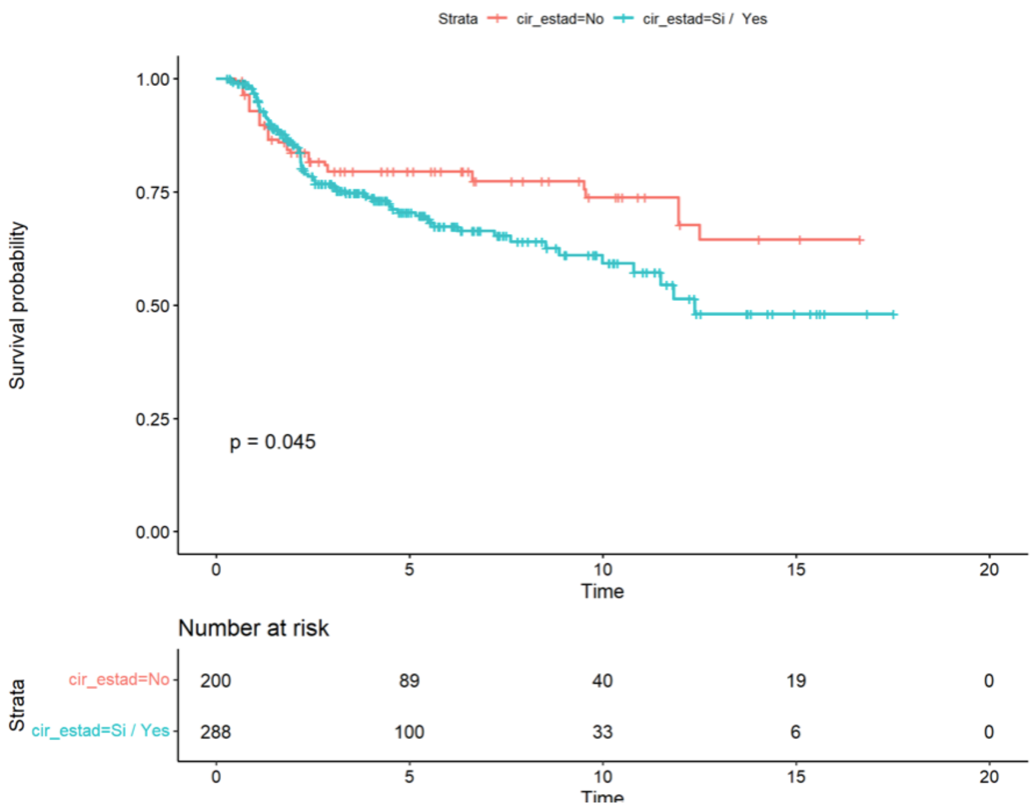

No PALND

##	time	n.risk	n.event	survival	std.err	lower 95% CI	upper 95% CI
##	2	139	31	0.837	0.0269	0.785	0.891
##	5	89	6	0.795	0.0305	0.738	0.857
##	10	40	4	0.738	0.0401	0.664	0.821

PALND

##	time	n.risk	n.event	survival	std.err	lower 95% CI	upper 95% CI
##	2	202	39	0.854	0.0217	0.812	0.897
##	5	100	31	0.705	0.0306	0.647	0.768
##	10	33	10	0.593	0.0430	0.514	0.684

1.2. Cancer specific survival

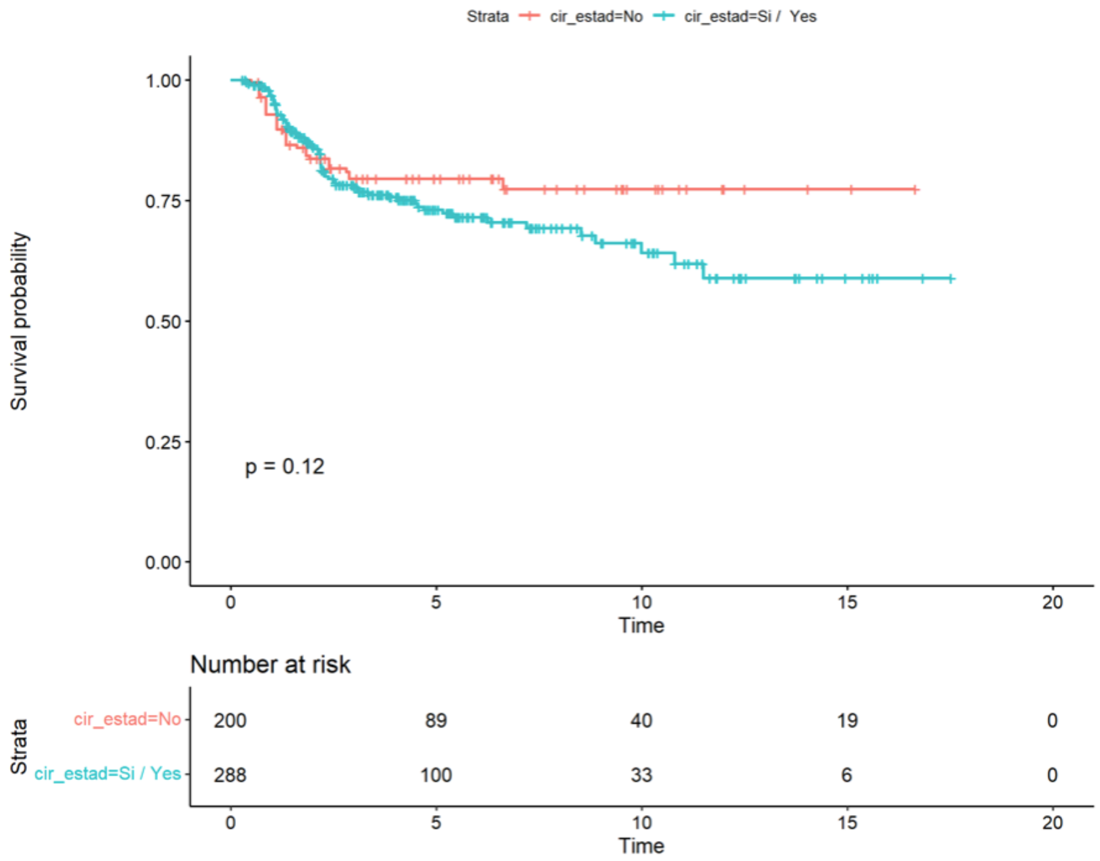


```
## NoPALND
##  time n.risk n.event survival std.err lower 95% CI upper 95% CI
##     2    139      31    0.837  0.0269        0.785        0.891
##     5     89       6    0.795  0.0305        0.738        0.857
##    10     40       2    0.774  0.0331        0.712        0.842
##
## PALND
##  time n.risk n.event survival std.err lower 95% CI upper 95% CI
##     2    202      37    0.861  0.0213        0.820        0.904
##     5    100      27    0.731  0.0297        0.675        0.791
##    10     33       7    0.642  0.0424        0.564        0.731
```

1.3. Disease free survival

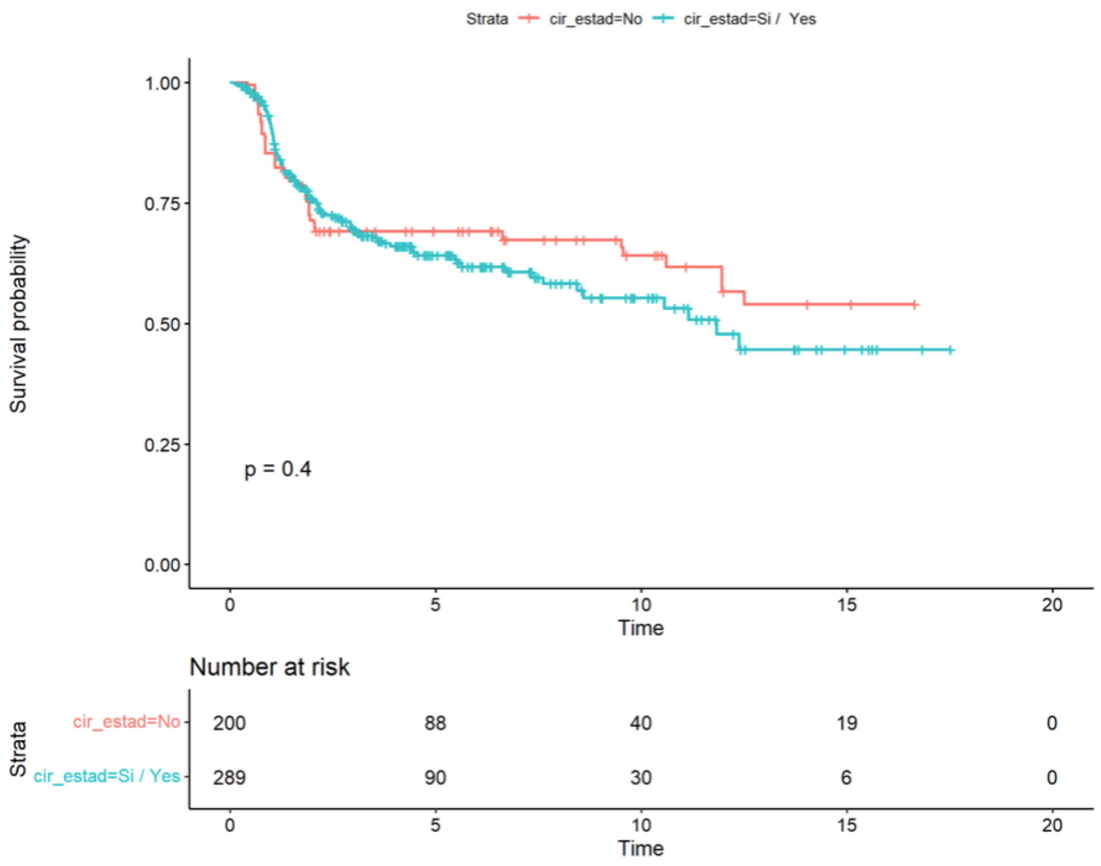


```
## NoPALND
##  time n.risk n.event survival std.err lower 95% CI upper 95% CI
##     2    127      55    0.714  0.0328        0.653        0.781
##     5     88       4    0.692  0.0336        0.629        0.761
##    10     40       4    0.642  0.0398        0.569        0.725
##
## PALND
##  time n.risk n.event survival std.err lower 95% CI upper 95% CI
##     2    180      65    0.759  0.0261        0.710        0.812
##     5     90      24    0.641  0.0315        0.582        0.706
##    10     30       8    0.554  0.0404        0.480        0.639
```

2. FIGO III

2.1. Overall survival

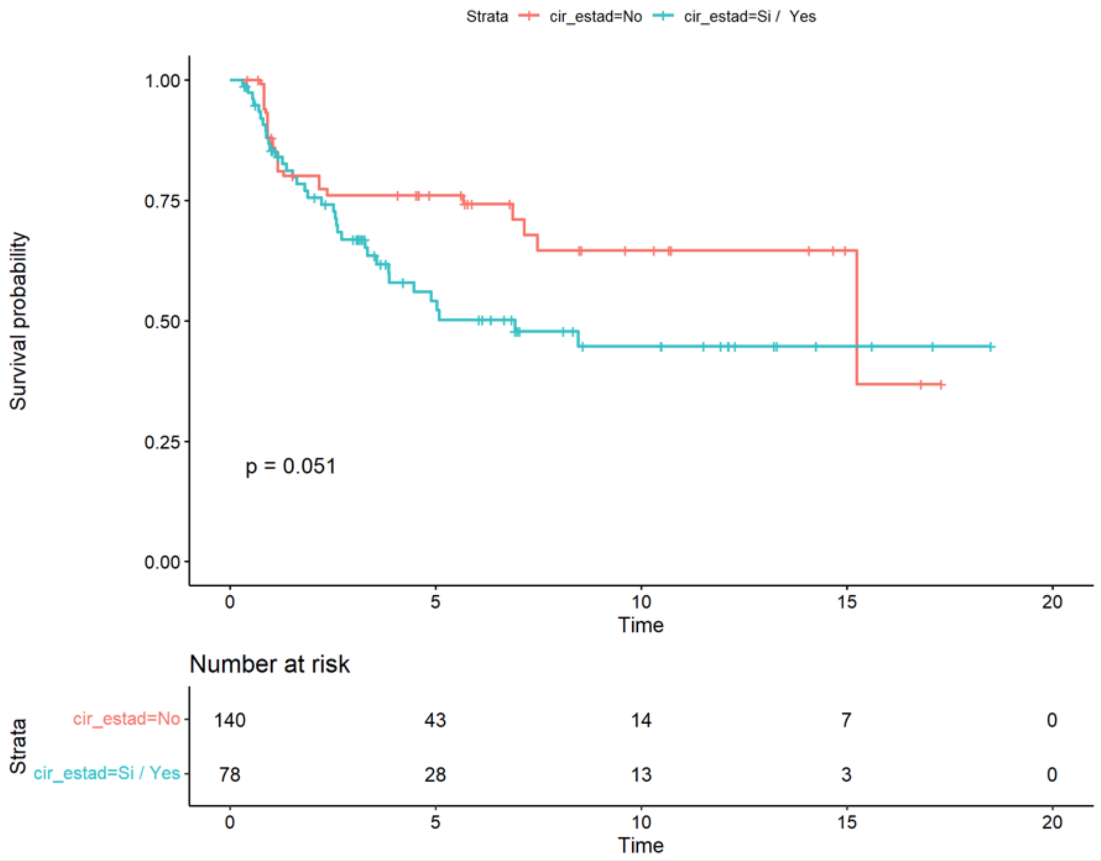


```
## NoPALND
##  time n.risk n.event survival std.err lower 95% CI upper 95% CI
##     2     59      24    0.802  0.0368        0.732        0.877
##     5     43       3    0.761  0.0418        0.683        0.847
##    10     14       4    0.646  0.0650        0.530        0.787
##
## PALND
##  time n.risk n.event survival std.err lower 95% CI upper 95% CI
##     2     54      18    0.756  0.0501        0.664        0.861
##     5     28      13    0.541  0.0626        0.431        0.679
##    10     13       4    0.447  0.0682        0.331        0.603
```

2.2. Cancer specific survival

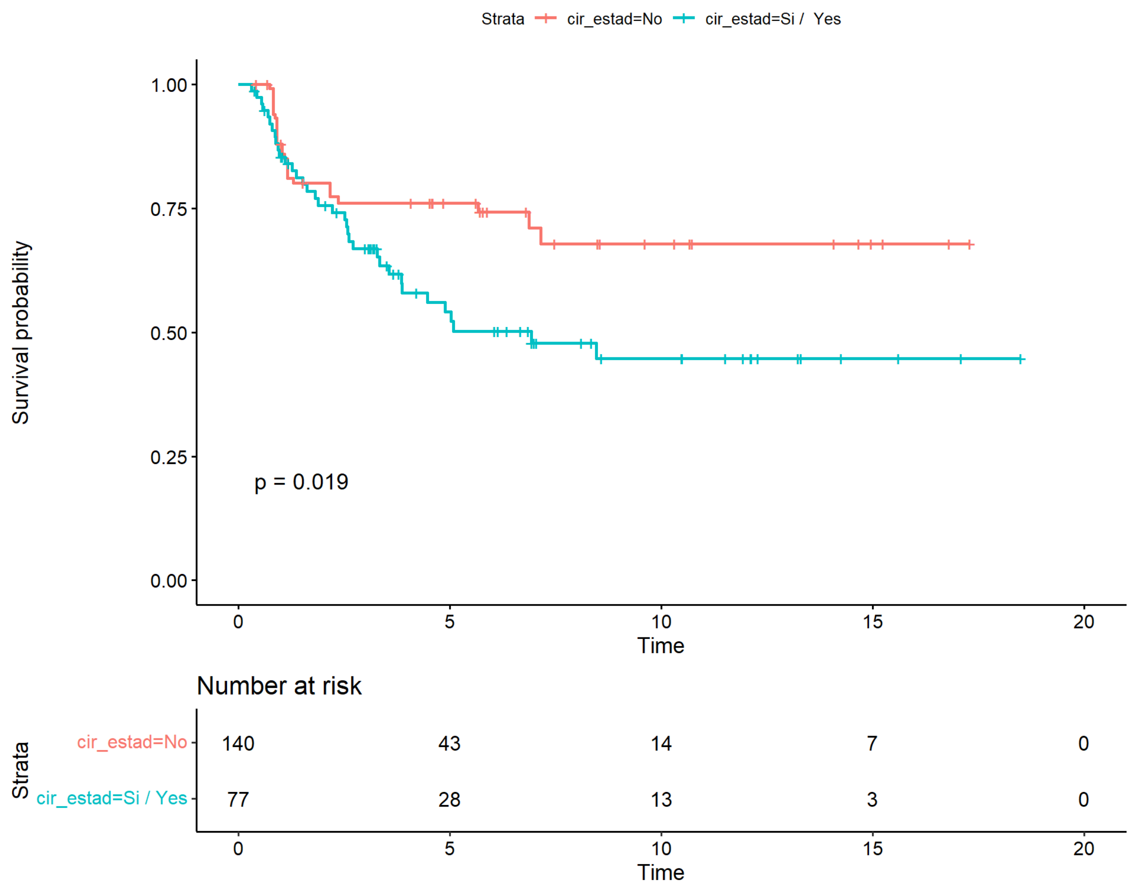


```
## NoPALND
##  time n.risk n.event survival std.err lower 95% CI upper 95% CI
##     2     59      24    0.802  0.0368        0.732        0.877
##     5     43       3    0.761  0.0418        0.683        0.847
##    10     14       3    0.678  0.0597        0.571        0.806
##
## PALND
##  time n.risk n.event survival std.err lower 95% CI upper 95% CI
##     2     54      18    0.756  0.0501        0.664        0.861
##     5     28      13    0.541  0.0626        0.431        0.679
##    10     13       4    0.447  0.0682        0.331        0.602
```

2.3. Disease free survival

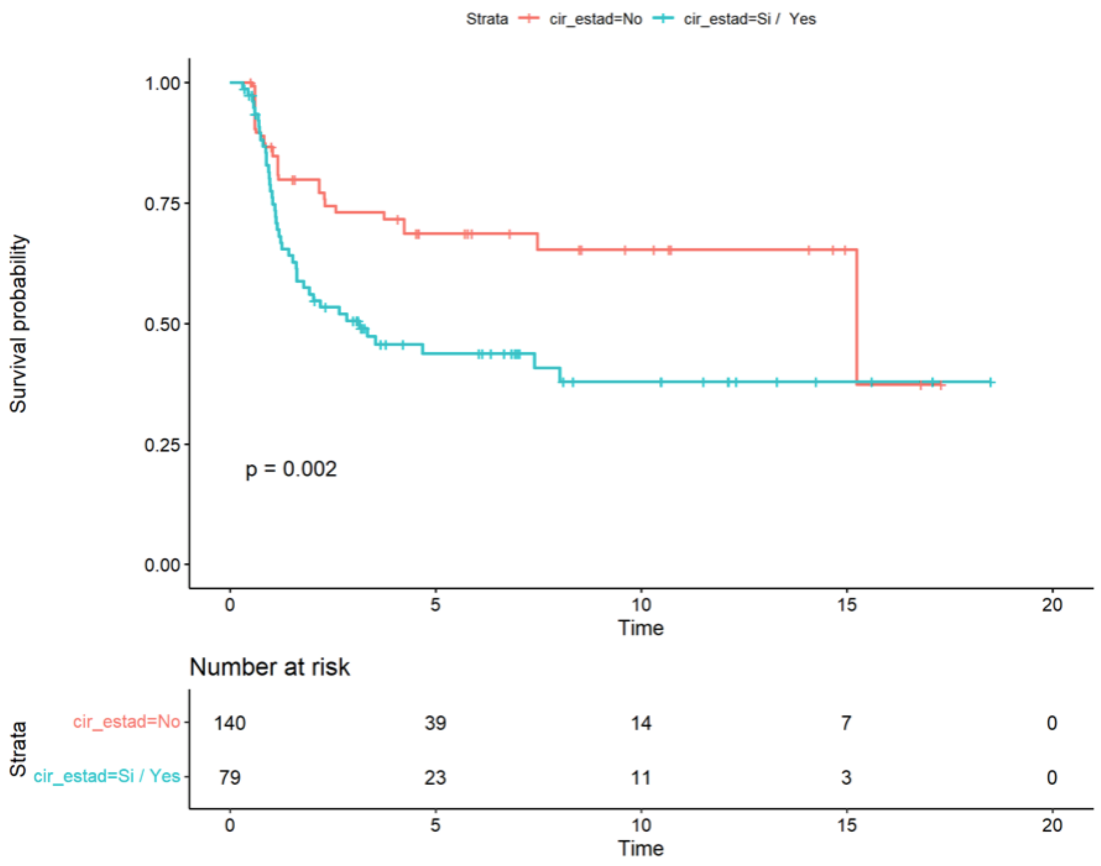


```
## NoPALND
##  time n.risk n.event survival std.err lower 95% CI upper 95% CI
##     2     58      25    0.799  0.0364        0.731        0.874
##     5     39       8    0.687  0.0484        0.598        0.788
##    10     14       1    0.654  0.0561        0.553        0.774
##
## PALND
##  time n.risk n.event survival std.err lower 95% CI upper 95% CI
##     2     42      33    0.561  0.0573        0.459        0.685
##     5     23       8    0.438  0.0594        0.335        0.571
##    10     11       2    0.379  0.0642        0.272        0.529
```
